# Regional Agriculture and Food Systems Amid the COVID-19 Pandemic: The Case of the Near East and North Africa Region

**DOI:** 10.3390/foods13020297

**Published:** 2024-01-17

**Authors:** Hamid El Bilali, Tarek Ben Hassen

**Affiliations:** 1 International Centre for Advanced Mediterranean Agronomic Studies (CIHEAM-Bari), Via Ceglie 9, 70010 Valenzano, Bari, Italy; elbilali@iamb.it; 2 Program of Policy, Planning, and Development, Department of International Affairs, College of Arts and Sciences, Qatar University, Doha P.O. Box 2713, Qatar

**Keywords:** coronavirus, bibliometrics, food security, sustainable agriculture, sustainable food system, resilience, Maghreb, MENA, NENA, Arab

## Abstract

The COVID-19 pandemic affected agri-food systems worldwide. However, while the impacts differed from one country/region to another, the scholarly literature seems to focus on developed countries in the Global North. Consequently, this review scrutinizes the literature on the pandemic’s impacts in the Near East and North Africa (NENA) region. A search on the Web of Science in March 2023 generated 334 documents, of which 151 were eligible for inclusion in the systematic review. According to the bibliometric analysis, the most active nations are Saudi Arabia, Egypt, Jordan, the United Arab Emirates, and Lebanon. In general, the coverage of studies is better in the Gulf region than in the less developed countries of North Africa and those suffering from wars (cf. Libya, Syria, and Yemen). Studies generally focus on crop production and the downstream food chain (cf. distribution and consumption). While the pandemic influenced every pillar of food security, this research concentrates on access and utilization. Meanwhile, the lion’s share of the literature deals with the pandemic’s socio-economic effects, especially those linked to food (in)security and health. The pandemic, which laid bare the agri-food system vulnerabilities, should be seized to foster the transition towards more resilient agri-food systems in the NENA region.

## 1. Introduction

In March 2020, the World Health Organization (WHO) declared the coronavirus disease 2019 (COVID-19), which is triggered by the Severe Acute Respiratory Syndrome Coronavirus 2 (SARS-CoV-2), a pandemic [1]. As of November 2023, the COVID-19 pandemic has affected every country and region, resulting in over 771 million reported cases and approximately 7 million fatalities [2]. The spread of the coronavirus (cf. confirmed cases), as well as the fatalities caused by it, varies by country/region [3,4,5,6,7,8]. In this regard, the Near East and North Africa (NENA) region was no exception. Indeed, the pandemic affected the economies of the NENA countries, e.g., Saudi Arabia [9]. Impacts on the economies had implications, inter alia, for food security [5]. The SARS-CoV-2 was not the first coronavirus that the Middle East region had to face since it was even the origin of another coronavirus named Middle East Respiratory Syndrome Coronavirus (MERS-CoV). Still, it was by far the most impactful. The confirmed cases, as well as the deaths, have been relatively high in the NENA region (Table 1). Indeed, NENA countries have been severely hit by the pandemic; the number of confirmed cases was higher than one million in several countries, namely Iraq, Jordan, Morocco, Lebanon, Tunisia, and the United Arab Emirates [2]. Despite this, the number of vaccines delivered and people immunized across the NENA area has been high, with respect to the total population [10], except for countries impacted by political instability and conflict, such as Yemen, Iraq, Libya, and Syria (Table 1). 

Aside from its health-related consequences, the pandemic caused an unprecedented worldwide crisis with far-reaching, complex consequences [11,12,13]; it caused an unprecedented global economic and financial crisis [14] and slowed down the attainment of the Sustainable Development Goals (SDGs) [15]. Moreover, it disrupted the global agri-food system [7,13,16,17,18,19,20,21,22]. Indeed, the pandemic affected food system activities and outcomes [7,22,23,24,25,26,27,28,29], with far-reaching and multidimensional implications regarding food security [21,23,27]. 

In this context, the current systematic review examines the consequences of the pandemic on agricultural and food systems in NENA countries. This study focuses on examining the bibliometrics and geographical aspects of this research topic while also analyzing its coverage of important subjects such as food security and food system sustainability.

## 2. Literature Review

The impacts of the pandemic on food systems and food security have been induced by the virus control measures—such as lockdowns; home quarantines; and social distancing—imposed by governments [20,30]. The effects of the pandemic were evident not just in developing nations but also in developed countries [5,27]. Effects on food security were especially severe in emerging low- and middle-income nations, affecting all pillars (namely availability, access, utilization, and stability) [31]. Furthermore, according to many studies [31,32,33,34,35], the COVID-19 pandemic affected consumers’ eating, shopping, and interaction with food. Indeed, the pandemic induced changes in food purchase modes across the globe; for instance, many people reduced the number of shopping trips during the lockdown and opted to buy more on each trip to minimize their exposure to the virus during store visits. Another shift was a move towards healthier diets, as consumers became more aware of the need to maintain a healthy immune system during the pandemic. Furthermore, there has been an increase in culinary capabilities as more people stayed at home and had more time to experiment with new recipes and cooking techniques. Finally, there were also changes in the generation and management of household food waste.

In the pre-COVID-19 period, agriculture played an important role in many NENA countries, and the region faced a daunting challenge in achieving food and nutrition security for its population. Data from the World Bank [36], pertaining to the period before COVID-19, confirm that the contribution of the primary sector (viz., agriculture, forestry, and fishing) to the gross domestic product (GDP) in the Arab World (cf. the NENA region) were just 4.88% in 2019, but it varied a lot from one country to another. Indeed, it ranged from less than 1% in many Gulf countries (viz., Bahrain, Kuwait, Qatar, and the UAE) to 20.16% in Sudan, 21.65% in Mauritania, and 40.74% in Syria (Table 2). Furthermore, as of 2019, employment in agriculture was 17.84% in the Arab World (cf. NENA), showing the high socio-economic importance of the primary sector; it ranged from less than 5% in Gulf countries (viz. Bahrain, Kuwait, Oman, Qatar, Saudi Arabia, and UAE), Lebanon, and Jordan to more than 30% in Mauritania, Morocco, Sudan, and Yemen. Before the onset of the pandemic, the NENA region already faced considerable challenges regarding food insecurity and malnutrition (Table 2). Indeed, the incidence of undernourishment was high in many NENA countries; in the period 2017–2019, it ranged from less than 2.5% in Algeria, Kuwait, and Tunisia to 11.9% in Mauritania, 12.4% in Sudan, and 23.7% in Iraq. The severity of the problem becomes more apparent and concerning when taking into account the rate of moderate or severe food insecurity, ranging from 12.3% in Kuwait to 44.8% in Mauritania and even as high as 48.9% in Sudan within the same time frame. Between 2017–2019 (pre-COVID-19 period) and 2020–2022 (COVID-19 period), the prevalence of undernourishment rose in many countries throughout the region, such as Egypt, Morocco, and Tunisia.

Meanwhile, moderate or severe food insecurity increased over the same period in Libya, Mauritania, Sudan, and Tunisia. Furthermore, the food security situation in NENA countries is vulnerable, as they rely heavily on imports to meet their growing food needs. For instance, the cereal import dependency ratio is high in all NENA countries (Table 2). Indeed, all NENA countries are net importers of cereals—a basic, staple food in the diets of their populations. The cereal import dependency ratio ranged in the period 2016–2018 from 23.60% in Sudan to 100% in Jordan and the UAE, which means that both countries import all the cereals they consume. This high reliance on cereal imports to meet domestic demand makes NENA countries particularly vulnerable to supply chain disruptions and global food price volatility, especially in the case of geopolitical crises and shocks [37,38,39].

**Table 2:** Agriculture and Food (in)security in NENA Countries.

Country	Agriculture, Forestry, and Fishing, Value Added (% of GDP)—2019	Employment in Agriculture (% of Total Employment)—2019	Prevalence of Undernourishment (% Population)		Prevalence of Moderate or Severe Food Insecurity (% Population)		Cereal Import Dependency Ratio 2016–2018 (%)
			2017–2019	2020–2022	2017–2019	2020–2022	
Algeria	12.34	9.94	2.8	<2.5	17.6	19.4	70.80
Bahrain	0.28	0.99	n.a.	n.a.	n.a.	n.r.	n.a.
Egypt	10.70	21.15	4.7	7.2	34.2	28.5	47.80
Iraq	3.77	20.06	23.7	16.3	n.a.	n.r.	56.80
Jordan	4.37	3.36	8.5	n.a.	n.a.	n.r.	100.0
Kuwait	0.38	2.01	<2.5	<2.5	12.3	10.9	98.80
Lebanon	3.17	3.82	5.7	n.a.	n.a.	36.5	99.00
Libya	4.09	16.94	n.a.	8.4	35.9	39.8	n.a.
Mauritania	21.65	30.64	11.9	8.7	44.8	53.7	n.a.
Morocco	10.84	33.94	4.3	6.3	25.9	n.r.	56.90
Oman	1.99	4.23	7.8	2.8	n.a.	n.a.	93.60
Qatar	0.26	1.20	n.a.	n.a.	n.a.	n.a.	n.a.
Saudi Arabia	2.56	3.48	4.8	3.8	n.a.	n.r.	95.60
Sudan	20.16	40.69	12.4	11.9	48.9	51.8	23.60
Syria	40.74	13.06	n.a.	27.8	n.a.	n.a.	n.a.
Tunisia	9.77	14.29	<2.5	3.0	20.0	28.5	65.50
UAE	0.75	2.24	3.1	<2.5	n.a.	9.8	100.00
Yemen	n.a.	30.00	n.a.	34.5	n.a.	67.2	n.a.
Source	World Bank [36]	World Bank [40]	FAO et al. [41]	FAO et al. [42]	FAO et al. [41]	FAO et al. [42]	United Nations Statistics Division [43]

n.a. = data not available; n.r. = not reported.

Referring to politically fragile countries (e.g., Lebanon, Sudan, and Yemen) in the context of the ongoing conflict in Ukraine, Al-Saidi [44] proposes that a combination of political-economic instability, constrained domestic agriculture, and inadequate reliable grain reserves have exacerbated the existing food crisis in certain countries. Indeed, the pandemic exacerbated pre-COVID-19 crises in several countries of the MENA region [45], such as Syria [46], Iraq [46], Yemen [47], Palestine [48], and Lebanon [49]. In this respect, the Arab Forum for Environment and Development [50] suggests that existing issues have made Arab nations much more exposed to the regional implications of both the COVID-19 pandemic and the conflict in Ukraine. For example, the area was already dealing with food insecurity as a result of several issues, such as growing populations, droughts caused by climate change, and internal conflicts. This was worsened by the region’s lack of food self-sufficiency and reliance on imports. Arab nations have also traditionally had high water deficits, with water demand levels exceeding the growth of water resource supply rates. Meanwhile, Ben Hassen et al. [48] found that the pandemic affected the food security status in the Palestinian territories. Likewise, multiple crises (viz., COVID-19, the economic crisis, and the Beirut port explosions) increased the prevalence of food insecurity in Lebanon [51]. Some studies even posit that the pandemic slowed down the realization of the Sustainable Development Goals (SDGs) in several NENA countries, e.g., Morocco [52].

Only a few previous reviews addressed the COVID-19 pandemic in the NENA region. However, they were generally old and/or had partial coverage from both geographical and topical/thematic points of view (Table 3). As a result, no recent systematic review has shed light on the studies tying the pandemic to food and agriculture throughout the NENA region. 

## 3. Materials and Methods

The geographical coverage of the present study is similar to that of the FAO regional office for the NENA region [57], consisting of 18 countries: Algeria, Bahrain, Egypt, Iraq, Jordan, Kuwait, Lebanon, Libya, Mauritania, Morocco, Oman, Qatar, Saudi Arabia, Sudan, Syria, Tunisia, the United Arab Emirates, and Yemen. 

This systematic review [58,59] draws upon a search performed on the Web of Science (WoS) on 17 March 2023, using the following string: (“COVID-19” OR COVID-19 OR Coronavirus OR “SARS-CoV-2”) AND (“agricultur*” OR agro OR food) AND (“Near East” OR “Middle East” OR “West* Asia” OR “North* Africa” OR Maghreb OR “East* Mediterranean” OR “South* Mediterranean” OR Arab OR Gulf OR Algeria OR Bahrain OR Egypt OR Iraq OR Jordan OR Kuwait OR Lebanon OR Libya OR Mauritania OR Morocco OR Oman OR Qatar OR Saudi OR Sudan OR Syria OR Tunisia OR “United Arab Emirates” OR UAE OR Yemen). The search returned 334 documents that were assessed for eligibility based on three criteria: geographic location (i.e., the document focused on at least one country in the NENA region), thematic relevance (i.e., the document addressed *both* COVID-19 and agriculture/food topics), and document type (i.e., only research articles, book chapters, or conference papers were included; editorials and literature reviews were excluded). 

The selection of eligible documents is described in Table 4. First, 21 documents published before the outbreak of SARS-CoV-2 (cf. COVID-19) at the end of 2019 were excluded; indeed, they deal with other coronaviruses, especially the Middle East Respiratory Syndrome Coronavirus (MERS-CoV). Following the screening of the document titles, 17 were deemed ineligible because they did not refer to NENA; documents encompassing larger geographical areas (e.g., Africa, Asia, and the Mediterranean) or those with no indication of geographical coverage in the title were retained for the subsequent selection stages. An additional 139 documents were excluded after analyzing abstracts since they did not meet at least one of the eligibility criteria. For instance, some documents do not deal with COVID-19/SARS-CoV-2 but other coronaviruses such as MERS-CoV or Severe Acute Respiratory Syndrome Coronavirus (SARS-CoV). Documents analyzing the health effects of the pandemic or vaccines without any reference to food were excluded. Likewise, studies dealing with the efficacy of some drugs (e.g., Remdesivir) or plant-based treatments in in vitro tests were discarded. However, those describing the relationships between food intake and the immune system were considered. For instance, some studies refer to the Food and Drug Administration (FDA) in the United States or food agencies in other countries while dealing with the efficacy of some vaccines. Additionally, 7 ineligible documents were excluded after a detailed review of full-texts, which included reviews [5,53,54,55,56].

Consequently, 151 documents were eligible and were included in the systematic review (Appendix A), consisting of 149 articles and 2 proceeding papers.

The selected articles are generally based on cross-sectional online surveys. Whereas most of the studies focus on adults in general (especially those referring to convenient, voluntarily-recruited online respondents), others address specific socio-economic groups and categories such as children, pregnant and lactating women, as well as people affected by specific diseases and illnesses (Table 5).

The bibliometrics and themes covered were both considered in the examination of the chosen documents. This paper examines several aspects, including bibliographical metrics, research geographies, agricultural subsectors, food chain segments, food security, and sustainability (Table 6). 

## 4. Results and Discussion

This section first analyzes the bibliographical metrics and the geography of this research field (Section 4.1), then sheds light on the agriculture subsectors and food chain stages addressed in the scholarly literature (Section 4.2), before moving on to the impacts of the pandemic on food security and its different pillars (Section 4.3) as well as its impacts on sustainability dimensions (Section 4.4).

### 4.1. Bibliometrics and Research Geography

The first research publication that examined the consequences of the COVID-19 pandemic on agriculture and food systems in the NENA region appeared in June 2020. There was a time gap of many months between the virus’s spread and the publication of the initial findings. Therefore, scientific data were lacking. This highlights that time is needed to produce robust evidence and publish it (cf. the peer-review process), but also questions the efficacy of research in dealing with emergencies and crises. Therefore, there is a need to seriously reflect on how research and the scientific community, with the solid and robust knowledge they can produce, can be timelier and more useful in dealing with future crises and shocks, such as pandemics. Indeed, the lack of scientific evidence and science-based knowledge favors the dissemination and rooting of misconceptions, wrong and speculative information, and even ‘fake news,’ which might result in detrimental effects on the health of the population. 

The *annual output* of publications is relatively high and has been increasing from one year to another, thus reaching its peak of 74 in 2022 (Appendix A). However, expecting a rise in the number of publications in 2023 would be somewhat speculative; on the one hand, it is always true that it takes time to see research results published, if any, but, on the other hand, it is clear that the pandemic is no longer in the spotlight and researchers might divert their interest and effort towards other topics, depending, inter alia, on funding opportunities.

**Figure 1:**
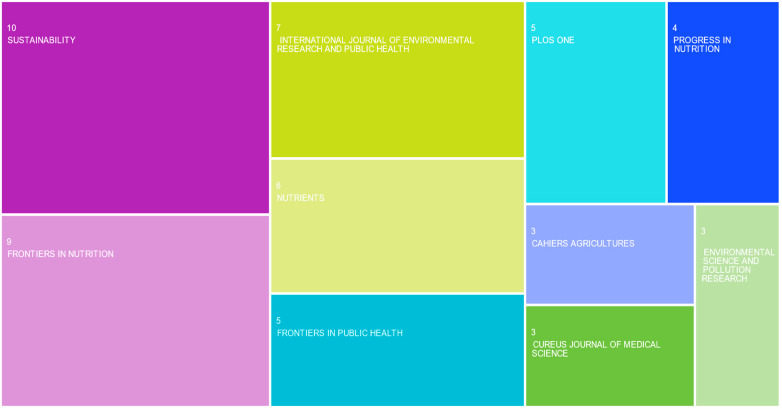
Publication titles and journals. Different colors show different journals. Numbers refer to the numbers of articles. Source: Authors’ elaboration based on data from the Web of Science database.

As for *publication titles* (Figure 1), the highest number of articles were published in *Sustainability* (10 articles, 6.62%), followed by *Frontiers in Nutrition* (9 articles, 5.96%), *International Journal of Environmental Research and Public Health* (7 articles, 4.64%), *Nutrients* (6 articles, 3.97%), and *Frontiers in Public Health* and *PLOS One* (5 articles, 3.31%, each). Nonetheless, the 151 papers describing the conclusions of the research on the effects of the pandemic on agri-food systems in the NENA area were published in 96 sources and journals, indicating no dominant publishing outlet. 

**Figure 2:**
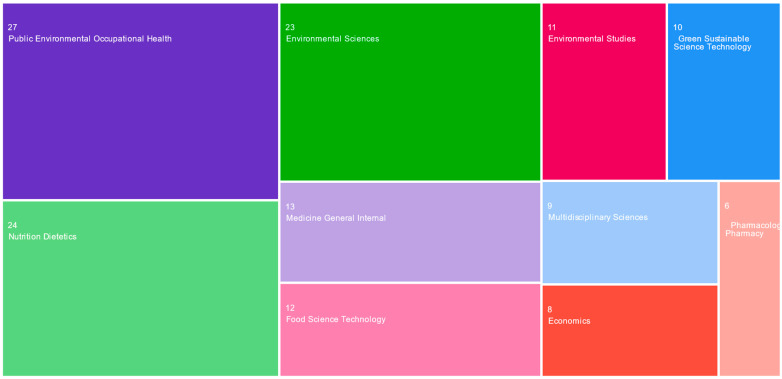
Research areas. Different colors show different areas. Numbers refer to the numbers of articles. Source: Authors’ elaboration based on data from the Web of Science database. Pharmacolog: Pharmacology.

Most of the selected articles are in this *research areas* of *Public environmental Occupational health* (27 articles, 17.88%), *Environmental sciences—Ecology* and *Nutrition dietetics* (24 articles, 15.89% each), *Science technology* (19 articles, 12.58%), *General internal medicine* (14 articles, 9.27%), *Business economics* (13 articles, 8.60%), *Food science technology* (12 articles, 7.94%), and *Agriculture* (10 articles, 6.62%) (Figure 2). Nevertheless, the 151 eligible articles can be categorized under 32 research fields (e.g., pharmacology, psychiatry, psychology, development studies, engineering, geography, computer science, biology, pediatrics, sociology, surgery, tropical medicine, veterinary sciences, and zoology), which demonstrate that this research field is multidisciplinary.

**Figure 3:**
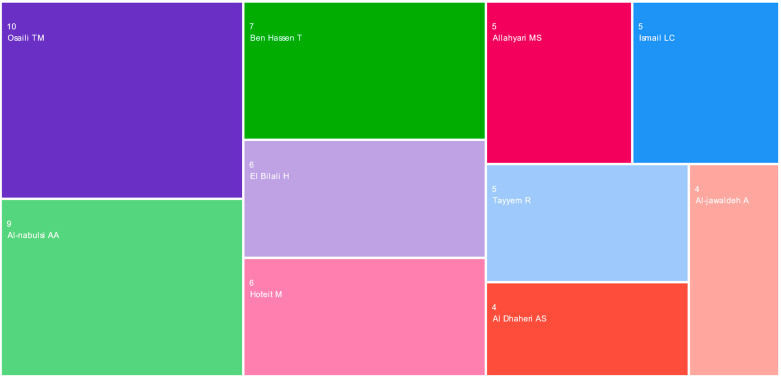
Authors. Different colors show different authors. Numbers refer to the numbers of articles. Source: Authors’ elaboration based on data from the Web of Science database.

According to the findings, the most notable and active *authors* in this research area are Tareq M. Osaili (10 articles/6.62%, Jordan University of Science and Technology), Anas A. Al-Nabulsi (9 articles/5.96%, Jordan University of Science and Technology), Tarek Ben Hassen (7 articles/4.64%, Qatar University), Hamid El Bilali (6 articles/3.97%, CIHEAM-Bari, Italy), and Maha Hoteit (6 articles/3.97%, Lebanese University) (Figure 3). Nonetheless, the 151 chosen publications were written by 719 experts, indicating that most interested researchers wrote a single paper. This implies a significant level of cooperation within this study field, which is surprising considering the relatively recent emergence of this particular research area.

**Figure 4:**
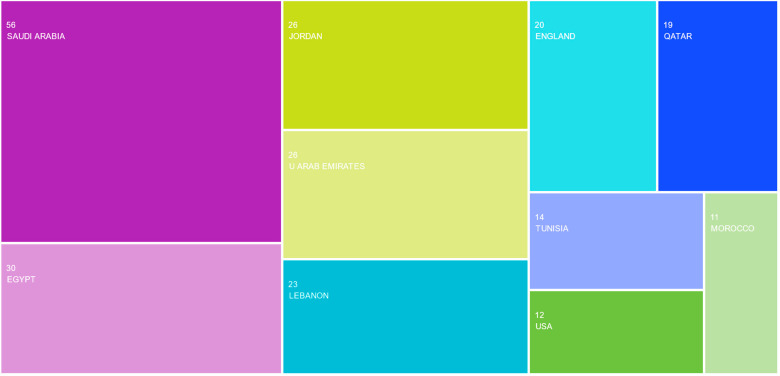
Affiliation countries. Different colors show different countries. Numbers refer to the numbers of articles. Source: Authors’ elaboration based on data from the Web of Science database.

The examination of affiliations reveals that the list of the most active *nations* is headed by Saudi Arabia (56 articles, 37.09%), Egypt (30 articles, 19.87%), Jordan, and the United Arab Emirates (26 articles, 17.22%, each), and Lebanon (23 articles, 15.23%) (Figure 4). The list of the top-ten affiliation countries includes eight NENA countries (viz., Saudi Arabia, Egypt, Jordan, the United Arab Emirates, Lebanon, Qatar, Tunisia, and Morocco), which might indicate the dynamism and strength of this research system in the region in general and the concerned countries in particular. The 151 chosen articles were written by academics and scientists from 56 different nations, which implies a strong collaboration between NENA researchers and those from abroad (especially from the UK and USA). 

**Figure 5:**
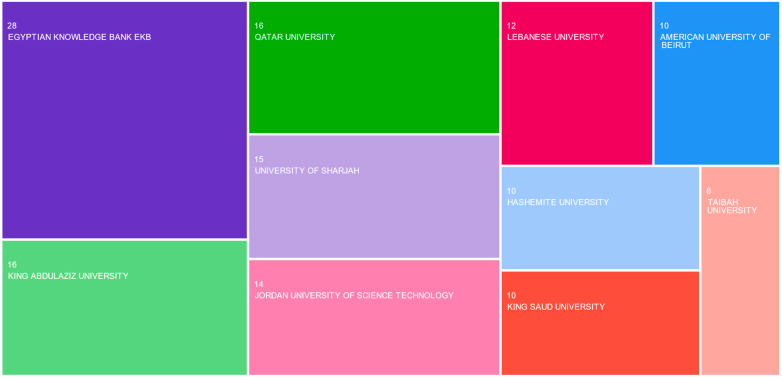
Affiliation institutions. Different colors show different institutions. Numbers refer to the numbers of articles. Source: Authors’ elaboration based on data from the Web of Science database.

Meanwhile, the analysis of affiliation *institutions* suggests that the most prominent research centers and universities in the research strand are located in NENA countries, namely the Egyptian Knowledge Bank (Egypt), King Abdulaziz University (Saudi Arabia), Qatar University (Qatar), University of Sharjah (UAE), Jordan University of Science and Technology (Jordan), and the Lebanese University (Lebanon) (Figure 5). This confirms the previous observation regarding the dynamism and vitality of the research systems in the concerned countries. In addition to the institutions already mentioned, other major institutions in the region are located in: Bahrain (University of Bahrain), Egypt (Al Azhar University, Mansoura University), Jordan (Hashemite University, University of Jordan, Al Balqa Applied University), Lebanon (American University of Beirut), Morocco (Moulay Ismail University of Meknes), Oman (Sultan Qaboos University), Palestine (Al Quds University), Saudi Arabia (King Saud University, Taibah University, King Faisal University, Princess Nourah Bint Abdulrahman University), and the UAE (United Arab Emirates University, Zayed University). The 151 publications chosen for this study were written by researchers affiliated with 338 universities and research institutes. This finding suggests a high level of cooperation within the scholarly community. 

The examination of the geographical characteristics of this research field reveals notable variations across the nations in the NENA region (Table 7). The majority of research examining the effects of the pandemic on agriculture and food systems was conducted in Saudi Arabia (46 articles), followed by Lebanon (15 articles), Jordan (12 articles), and Egypt (11 articles). While the high number of studies in Saudi Arabia and Egypt is somehow expected as these are large countries in the NEA region, the focus on Lebanon and Jordan, which are small countries, is rather surprising but might denote the dynamism of their national research systems. In general, the coverage of studies is better in rich countries of the Gulf region (except Bahrain and Oman) than in less developed countries of North Africa and those suffering from political instability or war (cf. Libya, Syria, and Yemen). Indeed, no single article specifically addresses Libya, Sudan, Mauritania, or Bahrain. Similarly, countries such as Algeria and Syria (1 article each), Yemen (2 articles), and Iraq and Oman (3 articles each) have been largely overlooked in this research field. 

Additionally, although there were no studies that analyzed the pandemic’s impacts on agri-food systems across the entire NENA region, there were some studies that looked at multiple countries in the region. For example, Tayyem et al. [85] analyze the impacts of the COVID-19 pandemic on food consumption patterns, dietary diversity, and body weight in 10 Arab countries (viz., Bahrain, Egypt, Jordan, Kuwait, Oman, Qatar, Saudi Arabia, the UAE, Lebanon, and Palestine). Meanwhile, Faour-Klingbeil et al. [94] examine how the pandemic influenced public views and attitudes surrounding food safety and health hazards in Jordan, Lebanon, and Tunisia. Al-Saidi and Hussein [95] offer a comprehensive assessment of the impacts and reactions to the COVID-19 pandemic on the water-energy-food (WEF) nexus in the Middle East region, specifically in Jordan, Lebanon, and the countries of the Gulf Cooperation Council. However, the most extensive study seems to have been performed by Abouzid et al. [96], who explored the effects of the pandemic on lifestyle behaviors in a survey of 5896 individuals from 17 countries in the MENA region (viz., Egypt, Jordan, the United Arab Emirates, Kuwait, Bahrain, Saudi Arabia, Oman, Qatar, Yemen, Syria, Palestine, Algeria, Morocco, Libya, Tunisia, Iraq, and Sudan). Another comprehensive study is that of Kilani et al. [97], who studied the impacts of lifestyle behaviors, such as food and diet choices, on university students’ mental health during lockdown and confinement policies related to the COVID-19 pandemic in the Levant region (viz. Jordan, Lebanon, Palestine, and Syria), the Arab Gulf region (viz. Bahrain, Iraq, Kuwait, Oman, Qatar, Saudi Arabia, and the UAE), the North Africa region (viz. Algeria, Egypt, Libya, Morocco, and Tunisia), and Yemen and Sudan. 

**Table 7:** Spatial Distribution of Research Examining the Effects of the COVID-19 Pandemic on Agriculture and Food Systems in the NENA Region. Source: Authors.

Country or Region (Articles Number)	Documents
Algeria (1)	Benmerzoug et al. [65]
Bahrain (0)	
Egypt (11)	Abu Hatab et al. [98]; Abu Hatab et al. [99]; Ali and Gad [100]; Batisha [101]; El-Haddad and Zaki [102]; Khabour and Hassanein [103]; Marzouk et al. [104]; Mohsen et al. [105]; Nour [106]; Selim and Eltarabily [107]; YahiaMarzouk and Jin [108]
Iraq (3)	Ahmed [86]; Al-Doori et al. [109]; Lafta and Mawlood [110]
Jordan (12)	Abualhaija and Shammout [111]; Almanasrah et al. [74]; Elsahoryi et al. [112]; Issa et al. [113]; Khamees et al. [114]; Olaimat et al. [115]; Olaimat et al. [116]; Osaili et al. [117]; Osaili et al. [83]; Osaili et al. [118]; Taybeh et al. [119]; Tayyem et al. [89]
Kuwait (5)	Al-Sejari and Al-Ma’Seb [120]; AlTarrah et al. [121]; Husain and Ashkanani [122]; Saleh [123]; Zainal et al. [124]
Lebanon (15)	Ben Hassen et al. [125]; Cheikh Ismail et al. [126]; Dimassi et al. [127]; El Khoury and Julien [128]; Fiddian-Qasmiyeh [129]; Hamade [130]; Hammoudi et al. [131]; Kharroubi et al. [132]; Al Kassaa et al. [87]; Chaiban et al. [133]; Gedeon et al. [67]; Hoteit et al. [69]; Kamaleddine et al. [70]; McCall et al. [49]; Nohra et al. [134]
Libya (0)	
Mauritania (0)	
Morocco (7)	Bossenbroek and Ftouhi [78]; Bouzidi and Abdellaoui [79]; El Bilali et al. [135]; Rachidi et al. [136]; Saidi et al. [137]; Saidi et al. [138]; Sraïri [139]
Oman (3)	Alazaiza et al. [140]; Ben Hassen et al. [141]; Mansour et al. [142]
Qatar (5)	Al-Abdi et al. [143]; Alah et al. [144]; Al-Mulla and Mahfoud [63]; Ben Hassen et al. [145]; Kaitibie et al. [146]
Saudi Arabia (46)	Abduljawad [147]; Abolfotouh et al. [148]; Aijehany and Allily [149]; Al Agha et al. [60]; Al Sadig et al. [150]; Alafif et al. [151]; Alamri et al. [152]; Aldhwayan and Alabdulkader [153]; Alfayez et al. [61]; Alghadir et al. [62]; Algheshairy et al. [73]; Alharthi [154]; Alhusseini and Alqahtani [155]; Alhusseini et al. [156]; Alkhalaf et al. [157]; Alkhaldy et al. [158]; ALkharashi [159]; Almousa and Alagal [160]; Al-Musharaf [75]; Al-Musharaf et al. [76]; Almutairi [161]; Alothman et al. [162]; Alotiby and Al-Harbi [163]; Alqurashi [164]; Alsuwailem et al. [165]; Alyami et al. [166]; Arfaoui and Alghafari [77]; Azazz and Elshaer [167]; Bakhsh et al. [168]; Braiji et al. [169]; Bushnaq et al. [170]; El-Akabawy et al. [80]; Elgammal et al. [171]; Hanbazaza [172]; Hanbazaza and Wazzan [68]; Hariri et al. [173]; Helal et al. [88]; Hesham et al. [174]; Jalal et al. [175]; Jawed et al. [176]; Mosli et al. [82]; Mumena [177]; Saaty and Aljadani [178]; Sobaih and Moustafa [179]; Sultan et al. [180]; Zakout et al. [181]
Sudan (0)	
Syria (1)	Alhaffar et al. [182]
Tunisia (5)	Ghali-Zinoubi [183]; Koussani and Khamassi [184]; Labidi [185]; Ragetlie et al. [186]; Turki et al. [187]
United Arab Emirates (8)	AlBlooshi et al. [188]; Ali et al. [189]; Radwan et al. [190]; Radwan et al. [191]; Sajwani et al. [72]; Samara et al. [192]; Sundarakani and Onyia [193]; Takshe et al. [194]
Yemen (2)	Butt et al. [66]; Rahmat et al. [47]
Near East/Middle East * (7)	Alalwan et al. [195]—Jordan and Saudi Arabia; Al-Saidi and Hussein [95]—Jordan, Lebanon, and the Gulf Cooperation Council; Ben Hassen et al. [6]—Lebanon, Palestine, Oman, and Qatar; Hoteit et al. [196]—Eastern Mediterranean; Tayyem et al. [84]; Woertz [197]—Arab Gulf states; Zuntz et al. [198]—Iraq, Jordan, Lebanon, Syria, and Turkey
North Africa ** (5)	Alouani et al. [199]—Maghreb (Tunisia, Algeria, and Morocco); Ben Hassen et al. [8]—Egypt, Morocco and Tunisia; Ben Khadda et al. [200]—Morocco, Algeria, and Tunisia; Ftouhi et al. [201]—Algeria and Morocco; Jouili and Elloumi [202]—Maghreb
NENA *** (8)	Abouzid et al. [96]—Egypt, Jordan, UAE, Kuwait, Bahrain, Saudi Arabia, Oman, Qatar, Yemen, Syria, Palestine, Algeria, Morocco, Libya, Tunisia, Iraq, and Sudan; Cheikh Ismail et al. [203]; El-Malah et al. [204]—Egypt and Saudi Arabia; Faour-Klingbeil et al. [205]—Lebanon, Jordan, and Tunisia; Faour-Klingbeil et al. [206]—Lebanon, Jordan, and Tunisia; Faour-Klingbeil et al. [94]—Jordan, Lebanon, and Tunisia; Hoteit et al. [81]; Kilani et al. [97]—Levant region (Jordan, Lebanon, Palestine, and Syria); Arab Gulf region (Bahrain, Iraq, Kuwait, Oman, Qatar, Saudi Arabia, and UAE); North Africa region (Algeria, Egypt, Libya, Morocco, and Tunisia); and Yemen and Sudan; Tayyem et al. [85]—Bahrain, Egypt, Jordan, Kuwait, Oman, Qatar, Saudi Arabia, United Arab Emirates, Lebanon, and Palestine
Global **** (6)	Ammar et al. [207]—Europe, North Africa, Western Asia, and the Americas; Bahatheg [64]—Saudi Arabia, Britain, and Turkey; Belton et al. [208]—Bangladesh, Egypt, India, Myanmar, and Nigeria; Mertens and Peñalvo [209]; Oakley et al. [71]—Ethiopia, Jordan, and Palestine; Pritchard et al. [210]—e.g., Democratic Republic of Congo (DRC), Bangladesh, and Yemen

* This group encompasses documents that analyze at least two Near East/Middle East countries. ** This group covers publications analyzing at least two North African countries. *** These publications examine a minimum of one country from North Africa and one from the Near East region. **** This group covers publications analyzing at least one country outside the NENA region.

### 4.2. Agriculture Subsectors and Food Chain Stages 

In regards to *agriculture* subsectors, the majority of the designated publications, particularly those focusing on shifts in food consumption habits and diets during the COVID-19 pandemic, did not mention specific subsectors. Publications examining a distinct agrifood subgroup usually analyze crop farming [100,107,137,138,184], whereas animal production/livestock [99,118,136,184] and, especially, fisheries/aquaculture [208] tend to receive less attention. As for crop production, considered crops include wheat [100], vegetables [137,138], cereals [189], and fruit [137,138]. Regarding animal production, studies regard poultry [99] and dairy cattle [118,136]. For example, Osaili et al. [118] examined the knowledge, perspectives, and behaviors of Jordan’s dairy sector employees in response to COVID-19 regulations and interventions. Only a few articles deal with aquaculture and fisheries; Belton et al. [208] shed light on the pandemic’s impacts on the value chains of aquatic foods. Some articles deal simultaneously with crop production and animal husbandry; for example, Koussani and Khamassi [184] explore the impacts of the pandemic on small-scale farmers, generally integrating plants and livestock, in Tunisia. 

The analysis of eligible studies indicates the pandemic impacted all levels of the food supply chain, including production, processing, transportation, distribution, and consumption. However, most of this research concentrated on the downstream or later phases of the food chain, namely distribution/retail, and consumption, as well as examining food waste. Concurrently, the early food chain phases (namely production) and the intermediary stages (such as handling, processing, and packing) are frequently neglected, as seen in Table 8. Specifically, much of the examined academic literature centers on the effects of the COVID-19 pandemic on diets, dietary behaviors, consumption patterns, and food security within the NENA region. 

The pandemic affected crop production and productivity in different ways. It made it more challenging for farmers, especially small-scale producers, to access inputs, which affected yield and production [100]. In Tunisia, Koussani and Khamassi [184] point out that the pandemic led to significant disruptions of agricultural operations, especially in supply chains for raw materials (such as crop protection products/pesticides, fertilizers, seeds, animal feed, etc.) as well as marketing channels. These issues were due to the closure of weekly markets selling perishable goods like leafy vegetables and small livestock. The pandemic also affected access to services such as extensions [107,186]. Selim and Eltarabily [107], in their study on the effects of the lockdown on small-scale farming in Egypt’s north-eastern Nile Delta, emphasize the issue that without agricultural extension opportunities, there is a noticeable gap in knowledge about enhancing farming methods and adopting smart irrigation systems, which are crucial for water conservation and boosting crop yields.

Only a few documents address the processing stage, and often in a marginal way. For instance, Yahia Marzouk and Jin [108] investigate how relational capital (RC) influences the robustness of small and medium-sized enterprises (SMEs) in the Egyptian food and beverage sector during the pandemic. 

The pandemic also affected the marketing and distribution of agri-food products. Several studies report increased recourse to food delivery and e-commerce for food procurement during the pandemic [6,73,77,130,135,156]. 

Numerous studies indicate that the COVID-19 pandemic adversely influenced dietary habits and food consumption patterns, particularly during lockdowns, leading to notable health consequences such as increased rates of overweight and obesity [62,80,157,160,170,187,190,199]. Alkhalaf et al. [157] found a reduction in the consumption of vegetables and an increase in sweet consumption among Saudi adults. Meanwhile, Braiji et al. [169], in their study focusing on Jeddah (Saudi Arabia), observed a notable decrease in the consumption of fast foods, like pizza and burgers, during the quarantine period, while the intake of snacks, sugars, and pastries saw a significant increase during the same time. Nevertheless, some studies point out some positive changes. For example, in their research on the eating habits of university students in the UAE during the COVID-19 period, Takshe et al. [194] discovered that a majority (67%) of the students surveyed appeared to have improved their dietary practices, opting for healthier life choices.

Moreover, several studies highlight that during the pandemic, there was an increased focus on health and healthy diets, particularly those believed to enhance and fortify the immune system [66,103,113,163,166,191]. Additional research addresses the issue of food security and insecurity during the pandemic period [47,69,112,116,132,198,209]. Other studies address the effects of the COVID-19 pandemic on food waste [137,140,167]. While some refer to a decrease in the generation of waste along the food chain [137,141], others underline an increase in food waste, especially at the household level [140]. Indeed, referring to the Omani context, Alazaiza et al. [140] discovered that a primary cause of the increased household waste generation during the lockdown was attributed to people spending more time at home.

Some studies take a more systemic, holistic approach and deal simultaneously with different food chain stages. They refer to the food supply chain [109,137,138,165,208] or value chain [197]. Referring to the fruit and vegetable supply chain in Meknes (Morocco), Saidi et al. [137] observed that the crisis prompted the emergence of new and more sustainable food supply methods, such as mutualized and local sourcing, as well as reduced food waste, leading the move towards sustainability. 

**Table 8:** Food chain stages (n = 151). Source: Authors.

Food Chain Stage *	Articles	Number and Share of Articles
Production	Abu Hatab et al. [99]; Ali and Gad [100]; Belton et al. [208]; Bossenbroek and Ftouhi [78]; Bouzidi and Abdellaoui [79]; Koussani and Khamassi [184]; Ragetlie et al. [186]; Saidi et al. [137]; Saidi et al. [138]; Selim and Eltarabily [107]; Sraïri [139]	11 (7.28%)
Processing	Abu Hatab et al. [98]; El-Haddad and Zaki [102]; Osaili et al. [83]; Osaili et al. [118]; Rachidi et al. [136]; YahiaMarzouk and Jin [108]; Zainal et al. [124]	7 (4.63%)
Marketing and distribution/retail (including transport, import/export, and logistics)	Abu Hatab et al. [98]; Al Sadig et al. [150]; Alalwan et al. [195]; Al-Doori et al. [109]; Algheshairy et al. [73]; Alhusseini et al. [156]; Ali et al. [189]; Almutairi [161]; Alsuwailem et al. [165]; AlTarrah et al. [121]; Belton et al. [208]; Ben Hassen et al. [145]; Ben Hassen et al. [6]; Ben Hassen et al. [141]; Dimassi et al. [127]; Elgammal et al. [171]; Ghali-Zinoubi [183]; Hamade [130]; Kaitibie et al. [146]; Saidi et al. [137]; Saidi et al. [138]; Sobaih and Moustafa [179]; Sundarakani and Onyia [193]; Taybeh et al. [119]; Zainal et al. [124]	25 (16.55%)
Consumption (including food services and food waste)	Abduljawad [147]; Abolfotouh et al. [148]; Abouzid et al. [96]; Ahmed [86]; Aijehany and Allily [149]; Al Agha et al. [60]; Al Kassaa et al. [87]; Al-Abdi et al. [143]; Alafif et al. [151]; Alah et al. [144]; Alamri et al. [152]; Alazaiza et al. [140]; AlBlooshi et al. [188]; Aldhwayan and Alabdulkader [153]; Alfayez et al. [61]; Alghadir et al. [62]; Algheshairy et al. [73]; Alhaffar et al. [182]; Alharthi [154]; Alhusseini and Alqahtani [155]; Alhusseini et al. [156]; Ali et al. [189]; Alkhalaf et al. [157]; Alkhaldy et al. [158]; ALkharashi [159]; Almousa and Alagal [160]; Al-Mulla and Mahfoud [63]; Al-Musharaf [75]; Al-Musharaf et al. [76]; Alothman et al. [162]; Alotiby and Al-Harbi [163]; Alouani et al. [199]; Alqurashi [164]; Alsuwailem et al. [165]; AlTarrah et al. [121]; Alyami et al. [166]; Ammar et al. [207]; Arfaoui and Alghafari [77]; Azazz and Elshaer [167]; Bahatheg [64]; Bakhsh et al. [168]; Belton et al. [208]; Ben Hassen et al. [145]; Ben Hassen et al. [125]; Ben Hassen et al. [6]; Ben Hassen et al. [141]; Ben Hassen et al. [8]; Ben Khadda et al. [200]; Benmerzoug et al. [65]; Braiji et al. [169]; Bushnaq et al. [170]; Butt et al. [66]; Cheikh Ismail et al. [126]; Cheikh Ismail et al. [203]; Dimassi et al. [127]; El Bilali et al. [135]; El Khoury and Julien [128]; El-Akabawy et al. [80]; Elgammal et al. [171]; El-Malah et al. [204]; Elsahoryi et al. [112]; Faour-Klingbeil et al. [206]; Faour-Klingbeil et al. [205]; Faour-Klingbeil et al. [94]; Gedeon et al. [67]; Ghali-Zinoubi [183]; Hammoudi et al. [131]; Hanbazaza [172]; Hanbazaza and Wazzan [68]; Hariri et al. [173]; Helal et al. [88]; Hesham et al. [174]; Hoteit et al. [81]; Hoteit et al. [69]; Hoteit et al. [196]; Husain and Ashkanani [122]; Issa et al. [113]; Jalal et al. [175]; Jawed et al. [176]; Kamaleddine et al. [70]; Khabour and Hassanein [103]; Khamees et al. [114]; Kharroubi et al. [132]; Kilani et al. [97]; McCall et al. [49]; Mertens and Peñalvo [209]; Mosli et al. [82]; Mumena [177]; Nohra et al. [134]; Olaimat et al. [115]; Olaimat et al. [116]; Osaili et al. [117]; Radwan et al. [190]; Radwan et al. [191]; Ragetlie et al. [186]; Rahmat et al. [47]; Saaty and Aljadani [178]; Saidi et al. [137]; Sajwani et al. [72]; Sobaih and Moustafa [179]; Sultan et al. [180]; Takshe et al. [194]; Taybeh et al. [119]; Tayyem et al. [89]; Tayyem et al. [84]; Tayyem et al. [85]; Turki et al. [187]; Zakout et al. [181]; Zuntz et al. [198]	108 (71.52%)

* Several documents address different stages of the food chain.

### 4.3. Impacts on Food Security and Nutrition 

The analysis of the eligible documents shows that the COVID-19 pandemic affected all four pillars of food security (viz., availability, access, use/utilization, and stability). Nevertheless, the scale of the impacts, as well as the level of coverage, change from one pillar to another (Table 9). In fact, most of the selected documents focus on food access and food utilization. 

Regarding *food availability*, the pandemic influenced domestic and global food production. The COVID-19 pandemic reduced domestic food production, leading to a subsequent reduction in food supply/availability. In some cases, access to inputs (e.g., fertilizers, pesticides, seeds) became more complicated, and their prices increased, which determined a decrease in their use with a consequent decrease in yield and productivity [100]. However, in their study in the Northeastern Nile Delta in Egypt, Selim and Eltarabily [107] noted that despite the costs of fertilizers and labor, as well as water availability near Port Said, being relatively unaffected by the lockdown, the overall income of small-scale farmers significantly decreased. Disruptions of global supply chains and markets influenced the trade and import of some products and inputs and, consequently, their availability in the domestic markets. Referring to Yemen, Rahmat et al. [47] observed that COVID-19 restrictions severely hindered food acquisition and the import of essential commodities.

Concerning *food access*, the analysis suggests that the COVID-19 pandemic influenced both economic accessibility/affordability and physical accessibility. Virus containment measures, such as lockdowns, impacted physical access to food in NENA countries. Nevertheless, these effects differed not only among NENA countries, based on the stringency and duration of the containment measures (cf. lockdown), but also among socio-economic groups. In particular, the poor [49], women [8,66,78,79,85,187], and children [65,66,67,69,182] seem to be more affected. Rahmat et al. [47], in their study on Yemen, caution that the combination of intense economic instability, worsened by the COVID-19 pandemic, ongoing conflict, and soaring food prices has brought the Yemeni population to the verge of famine, with women and children suffering particularly from malnutrition. Economic access was negatively affected due to the increase in food prices [211]. For instance, in Lebanon, the combined effects of the collapse of the Lebanese Pound (LBP) and COVID-19 led to a rise in the prices of foodstuffs by up to 50–60% in April 2020 concerning the pre-COVID period [212].

Moreover, the pandemic led to a significant decrease in purchasing power, primarily due to widespread job losses and its adverse impacts on various aspects of livelihoods. This downturn in purchasing power was exacerbated by the economic slowdown and disruptions in income sources, which further strained the financial capabilities of individuals and families, making it more challenging for them to afford essential goods and services, including food [47]. Referring to Syrian refugees in Middle Eastern countries, Zuntz et al. [198] state that during the spring of 2020, travel restrictions and disruptions in the supply chain led to displaced Syrian farm laborers losing their jobs and facing increased food poverty. In a review focusing on Tunisian agriculture, Elloumi [55] notes that the decline in the purchasing power of the most vulnerable groups in the population reduced their access to basic products. Ultimately, it was the reduction in international demand that most significantly impacted Tunisia’s primary agricultural sectors. In another review of childhood stunting in the Eastern Mediterranean region, Al Jawaldeh et al. [56] caution that the COVID-19 pandemic poses a risk of derailing efforts to combat stunting, as it affects both the accessibility and affordability of safe and nutritious foods, as well as the availability of crucial health services. 

However, the impacts of the pandemic on food access varied across the NENA region, with a marked difference between rich countries (especially those of the Gulf Cooperation Council) and other middle-income and low-income NENA countries. In a review focusing on the Gulf Cooperation Council region, Ben Hassen and El Bilali [5] discovered that while the pandemic affected how consumers interacted with food, it did not lead to panic buying and hoarding in the region. This finding contrasts with tendencies found around the globe throughout the pandemic. Furthermore, the GCC region’s population’s considerable buying power played a critical role in mitigating the economic consequences of the pandemic. Despite the global crisis, this financial resilience served to buffer the adverse effects on food and nutrition security in the region, ensuring that the population had greater access to food supplies and maintained a reasonably consistent level of nutrition and food security.

As for food *utilization*, the studies focus mainly on dietary diversity, diet quality, and food safety. The pandemic altered the nutritional value of diets by reducing the consumption of numerous health-promoting products [67,169], such as fruits and vegetables, as well as a surge in the use of unhealthy foods, such as sugar-sweetened drinks/beverages, pastries, sweets, and candies [54,169]. In a systematic review focusing on the impacts of the first wave of the pandemic, Mignogna et al. [54] discovered that there was an uptick in the consumption of suggested foods such as fruits, vegetables, legumes, cereals, and olive oil. However, there was a significant decline in fish consumption and a rise in that of dairy products. There was a decrease in items that should be consumed less often, such as red and processed meats. However, there was a noticeable rise in the consumption of unhealthy foods, such as snacks and sweets, during this period. This trend reflects a shift in dietary patterns, possibly due to the stress and lifestyle changes induced by the pandemic. Except for France, the data demonstrated better food quality across Europe, notably in Mediterranean nations.

In contrast, a shift to poorer nutrient patterns was noted in Colombia and Saudi Arabia. An analysis of eating behaviors indicated an increase in overall food intake, the number of daily meals, and snacking. The deterioration of consumption patterns, as well as the reduction of physical activity/exercise [160,169,173], especially during the lockdown, determined weight gains [85,173,187] and an increase in the prevalence of non-communicable diseases [49,156]. Nevertheless, there was also a rise in attention to and interest in health [103,113,163,166,191,204]. Meanwhile, people started paying more attention to the hygiene and safety of agri-food products [74,77]. However, referring to Jordanian female food handlers engaged in home-based food businesses, Osaili et al. [83] discovered that respondents had little understanding, unfavorable attitudes, and poor food safety practices.

Finally, regarding the *stability* pillar, many studies offer estimates and scenarios about the trajectory of food security in the context of the ongoing pandemic and its aftermath. For instance, Marzouk et al. [104] use system dynamics to model the effects of the COVID-19 pandemic on SDGs (including SDG 2, Zero Hunger) in Egypt and conclude that, based on governmental solid efforts to implement its 2030 goal, Egypt may achieve a declining proportion of food insecurity, reaching 3% in 2030. This percentage will continue to fall until it achieves complete sufficiency by 2050. Referring to food insecurity (FI) amid the pandemic and economic crisis in Lebanon, Kharroubi et al. [132] predict that there would be a more considerable prevalence of FI projections among females compared to men and among elderly persons in contrast to younger individuals.

**Table 9:** Impacts of the COVID-19 pandemic on food security in the NENA region. Source: Authors.

Food Security Dimension *	Topics Addressed	Documents
Food availability	Domestic food production and productivity	Abu Hatab et al. [99]; Ali and Gad [100]; Belton et al. [208]; Koussani and Khamassi [184]; Ragetlie et al. [186]; Saidi et al. [137]; Selim and Eltarabily [107]
	Food chains and markets	Al-Doori et al. [109]; Ali et al. [189]; Alsuwailem et al. [165]; Belton et al. [208]; Ghali-Zinoubi [183]; Hamade [130]; Kaitibie et al. [146]; Saidi et al. [138]; Sundarakani and Onyia [193]
Food access	Economic access and affordability	Alazaiza et al. [140]; Alhaffar et al. [182]; Ben Hassen et al. [145]; Ben Hassen et al. [125]; Ben Hassen et al. [141]; Ben Hassen et al. [8]; Dimassi et al. [127]; El Bilali et al. [135]; Oakley et al. [71]; Olaimat et al. [116]; Rahmat et al. [47]; Sobaih and Moustafa [179]; Tayyem et al. [85]; Zuntz et al. [198]
	Physical access	Abolfotouh et al. [148]; Ahmed [86]; Al Agha et al. [60]; Algheshairy et al. [73]; Alhusseini and Alqahtani [155]; Ali et al. [189]; Al-Musharaf et al. [76]; Alqurashi [164]; Alsuwailem et al. [165]; Ammar et al. [207]; Bahatheg [64]; Bakhsh et al. [168]; Belton et al. [208]; Ben Khadda et al. [200]; Cheikh Ismail et al. [126]; Cheikh Ismail et al. [203]; Faour-Klingbeil et al. [206]; Fiddian-Qasmiyeh [129]; Hanbazaza [172]; Jalal et al. [175]; Mansour et al. [142]; Mumena [177]; Nour [106]; Radwan et al. [190]; Rahmat et al. [47]; Saaty and Aljadani [178]; Saidi et al. [137]; Saidi et al. [138]; Sajwani et al. [72]; Sundarakani and Onyia [193]
Food utilisation	Diet quality and dietary diversity	Abduljawad [147]; Abolfotouh et al. [148]; Abouzid et al. [96]; Ahmed [86]; Aijehany and Allily [149]; Al Agha et al. [60]; Alafif et al. [151]; Alah et al. [144]; Alamri et al. [152]; AlBlooshi et al. [188]; Aldhwayan and Alabdulkader [153]; Alfayez et al. [61]; Alghadir et al. [62]; Algheshairy et al. [73]; Alharthi [154]; Alhusseini and Alqahtani [155]; Alhusseini et al. [156]; Alkhalaf et al. [157]; ALkharashi [159]; Almousa and Alagal [160]; Al-Musharaf [75]; Al-Musharaf et al. [76]; Alotiby and Al-Harbi [163]; Alqurashi [164]; Alyami et al. [166]; Ammar et al. [207]; Bahatheg [64]; Bakhsh et al. [168]; Ben Hassen et al. [145]; Ben Hassen et al. [125]; Ben Hassen et al. [6]; Ben Hassen et al. [141]; Ben Hassen et al. [8]; Ben Khadda et al. [200]; Braiji et al. [169]; Bushnaq et al. [170]; Cheikh Ismail et al. [126]; Cheikh Ismail et al. [203]; Dimassi et al. [127]; El Bilali et al. [135]; El Khoury and Julien [128]; El-Akabawy et al. [80]; Elsahoryi et al. [112]; Faour-Klingbeil et al. [206]; Gedeon et al. [67]; Hanbazaza [172]; Hanbazaza and Wazzan [68]; Hariri et al. [173]; Helal et al. [88]; Hesham et al. [174]; Hoteit et al. [81]; Hoteit et al. [69]; Hoteit et al. [196]; Husain and Ashkanani [122]; Issa et al. [113]; Jalal et al. [175]; Kamaleddine et al. [70]; Khabour and Hassanein [103]; Khamees et al. [114]; Kilani et al. [97]; Mertens and Peñalvo [209]; Mosli et al. [82]; Mumena [177]; Nohra et al. [134]; Olaimat et al. [116]; Radwan et al. [190]; Radwan et al. [191]; Saaty and Aljadani [178]; Sultan et al. [180]; Takshe et al. [194]; Taybeh et al. [119]; Tayyem et al. [89]; Tayyem et al. [84]; Tayyem et al. [85]; Turki et al. [187]
	Food safety	Abu Hatab et al. [98]; Almanasrah et al. [74]; AlTarrah et al. [121]; Arfaoui and Alghafari [77]; Dimassi et al. [127]; Faour-Klingbeil et al. [205]; Faour-Klingbeil et al. [94]; Olaimat et al. [115]; Osaili et al. [117]; Osaili et al. [83]; Osaili et al. [118]; Rachidi et al. [136]; Zakout et al. [181]
Stability	Food-related scenarios and projections	Al Sadig et al. [150]; Kharroubi et al. [132]; Marzouk et al. [104]; Woertz [197]

* Several documents address different food security dimensions.

### 4.4. Impacts on the Sustainability of Food Systems 

The body of evidence from the selected documents suggests that the COVID-19 pandemic affected all the dimensions of the sustainability of agri-food systems (Table 10). Nevertheless, as expected, the lion’s share of the scholarly literature deals with the socio-economic impacts of the pandemic, especially those linked to food (in)security and health, while environmental and political dimensions are less addressed. 

This study suggests that the *environmental* dimension is generally overlooked. For example, no study directly connects the COVID-19 pandemic to the conservation of biodiversity and/or the management of natural resources. Nevertheless, there are some exceptions. For instance, Abualhaija and Shammout [111] in their investigation into the impact of the pandemic lockdown on the quality of irrigation water in Jordan’s dams, including King Talal, Al-Kafrein, Al-Wehdeh, Kufranja, Wadi Al-Arab, and Zeqlab, found that there was an improvement in water quality and a decrease in pollution levels across all the dams studied during the COVID-19 lockdown. Selim and Eltarabily [107] compile learned lessons for water conservation amidst the COVID-19 lockdown in small-scale farming in the Northeastern Nile Delta (Egypt). Ftouhi et al. [201] point out that the pandemic promoted the adoption of agroecological practices in Algerian and Moroccan oases.

As for the *economic* dimension, the COVID-19 pandemic and the movement restrictions and limitations it determined affected socio-economic activities, including the primary sector (agriculture), which determined the loss of jobs and, consequently, income for many households. The effects have been especially dramatic for some vulnerable groups, such as refugees [49,198] and those living in countries suffering from civil wars, such as Yemen [47,66]. Furthermore, the pandemic affected the marketing of agri-food products, with an increase in online marketing and home delivery [73,156] and increased food prices in many NENA countries [47]. 

Regarding the *social* dimension, most studies address the impacts of the COVID-19 pandemic on health and food (in)security/(mal)nutrition. For instance, Hoteit et al. [196], in their analysis of the Eastern Mediterranean region, concluded that the COVID-19 crisis exposed the region’s lack of preparedness for a pandemic. They noted that while the aggressive containment strategy adopted by most countries in the region was crucial in preventing the spread of the virus, it resulted in a significant nutritional cost by leading to poor dietary diversity. Meanwhile, referring to the body weight and body mass index (BMI) of children in Constantine (Algeria), Benmerzoug et al. [65] underscore the risks stemming from changes in eating habits, including increased dietary intake and reduced physical activity, alongside a rise in sedentary behaviors, and how these factors contribute to the exacerbation of body weight gain and body mass index (BMI) increases. As shown in the previous section of this paper, the pandemic influenced all four pillars of food security in the NENA region.

Furthermore, the literature review shows that the pandemic increased vulnerability and poverty. This, in turn, led to a decrease in adherence to containment measures as the pandemic progressed since they were considered inappropriate, especially in the absence of adequate social protection measures. For instance, in Syria, Alhaffar et al. [182] highlight that lockdowns and the urge for self-isolation exemplified this dire situation. This happened despite the precarious reliance on daily earnings, the lack of income replacement subsidies, individual self-reliance, and poor trust and communication between communities and health authorities. Similarly, Nour [106] concluded that there was a deficiency in adherence to and participation in the stay-at-home rules in Damietta Governorate, Egypt. Sociodemographic factors impacted the public’s reaction to these directives, and a lack of confidence in government actions, community resources, and emergency services ensued. 

In general, the pandemic affected the quality of life and lifestyles [105,126,144,203]. Cheikh Ismail et al. [203] discovered that the lockdown imposed during the COVID-19 pandemic led to a range of lifestyle alterations, increased physical inactivity, and psychological issues (e.g., anxiety) among adults in the MENA region. In their study focusing on Dakahlia governorate in Egypt, Mohsen et al. [105] determined that the COVID-19 pandemic had a notable impact on the overall quality of life and personal safety of the public. The pandemic’s effects are differentiated by socio-economic groups and genders. Referring to Ethiopia, Jordan, and Palestine, Oakley et al. [71] reach the conclusion that the pandemic has worsened existing gender inequalities among adolescents in these diverse countries. Furthermore, they find that the current social safety nets are insufficient to fully mitigate these impacts, especially for the most vulnerable individuals. The pandemic and the measures taken to reduce the propagation of the virus also had some cascading effects. For instance, a study by Olaimat et al. [116] in Jordan underscores the negative consequences of COVID-19 restrictions on nutritional status, particularly among households experiencing food insecurity. These restrictions can exacerbate difficulties in accessing food due to economic challenges.

While many studies end up with some *policy* recommendations and suggestions, for instance, by calling for the inclusion of agri-food in the recovery plans and strategies [6], only very few deal with policy and governance. Studies dealing with the policy dimension generally address the implications of the virus containment measures and/or the effects of the measures taken by governments on the economy and population’s livelihoods [101,104], especially on food (in)security, and/or the adequacy of current agri-food policies in light of the lessons learned from the pandemic [139,197,202]. Referring to Maghreb countries, Jouili and Elloumi [202] caution that these crises have brought to light the vulnerabilities of food security dependent on international trade and the dangers of excessive import reliance. As a result, they advocate for reevaluating agricultural and food policy decisions with a greater emphasis on domestic food production. Meanwhile, as for governance, Alalwan et al. [195] analyze the effect of digital transformation and the increasing use of ICT during the COVID-19 pandemic on marketing governance within B2B (business-to-business) relationships in the Arab countries of Asia.

Some studies simultaneously address different *sustainability dimensions*. For instance, some articles analyze the effects of the pandemic on the implementation and achievement of the SDGs [101,104]. Marzouk et al. [104] examine the pandemic’s impacts on the achievement of SDGS 1 (No poverty), 2 (Zero hunger), 8 (Decent work and economic growth), and 13 (Climate action) in Egypt. Saidi et al. [137] refer to five dimensions (viz., ecological, economic, social, territorial, and food security) in their assessment of the sustainability of the supply chain of fruit and vegetables (SCF&V) in Meknes (Morocco). Referring to the social and economic impacts of the pandemic in the Iraqi context, Lafta and Mawlood [110] found that the suspension of educational activities presented the most tremendous social burden to people. At the same time, the rise in food prices and work stoppages were the primary causes of economic strain. 

Some scholars made *recommendations* to make the agri-food systems in the NENA region more sustainable and resilient to the COVID-19 pandemic and further shocks, stresses, and crises (Table 11). Different scholars stress the need to use the lessons learned from the pandemic to improve preparedness for future pandemics, crises, and shocks that can affect the agri-food system. In particular, some scholars call for strengthening social safety nets and protection policies [71,197]. Some scholars also call for paying more attention to the agri-food sector and food security issues in continuity plans and recovery strategies [6,209]. Mertens and Peñalvo [209] emphasize that response plans for COVID-19 in malnourished countries, which are at a heightened risk of fatal COVID-19, should prioritize food security, nutrition, and social protection measures. This is crucial to mitigating COVID-19 mortalities.

Further, policies must be evidence-based to be effective, and research is paramount in that respect. Following a study on food insecurity (FI) amidst the COVID-19 pandemic and economic crises in Lebanon, Kharroubi et al. [132] conclude that these frightening results demand emergency food security policies and evidence-based initiatives to alleviate the burden of numerous crises on the FI of Lebanese families and improve resilience to future shocks. Some scholars consider the pandemic an opportunity to rethink the agri-food sector and foster the transition towards more sustainable and resilient agri-food systems. In this respect, referring to the water-energy-food (WEF) nexus, Al-Saidi and Hussein [95] suggest that when it comes to resource-security concerns within the Water-Energy-Food (WEF) nexus, the COVID-19 stress test sparks discussions about the sufficiency of production value chains, which includes factors such as contingency and storage, diversification, and self-sufficiency. Furthermore, it raises concerns about the significance of cross-border integration in areas such as commerce/trade, globalization, and assistance. 

In general, several authors call for the reduction of import dependency and the diversification of import sources [189] to increase the resilience of the domestic food system. According to Ali et al. [189], the UAE has significantly relied on one or two sources for cereal imports, which are generally price-competitive, raising the danger of external cereal supply. The UAEs growing reliance on Russia as its primary supplier of cereals, as well as the consolidation of sources, represents a severe threat to food security. Reducing reliance on imports, which is a source of vulnerability to shocks on international markets, also calls for increasing domestic crop production; this option is considered especially relevant in North African/Maghreb countries [202]. 

Moreover, the experience of the COVID-19 pandemic shows clearly that containment measures should take into consideration the specific context and socio-economic situation of the population to be effective and not harm the livelihoods of local communities, especially the poor and those engaged in informal sectors [63,106,182]. This also implies adopting gender-sensitive approaches to empower women and reduce gender inequality [8,85,186].

The pandemic had adverse effects on the health of the population, and for that, it is necessary to improve its nutritional knowledge. In this respect, Alkhalaf et al. [157] highlight the significance of public health campaigns in enhancing the nutritional awareness of the population. These campaigns play a crucial role in educating individuals about the principles of healthy eating, promoting adherence to national dietary guidelines, and disseminating reliable and accurate information from authorized official sources. Furthermore, given the strong relationship between the pandemic and food safety, many scholars call for the training of agri-food sector workers on this matter [74,94,115]. They also put forward the need to raise the general public’s awareness of infection prevention measures and good hygiene practices [94,206]. 

The pandemic also underscored the need to adopt a holistic approach to dealing with agri-food systems. For instance, Ben Hassen et al. [6] highlight the interconnectedness of the dimensions of food systems and the need to address the challenges jointly. According to their assertion, several challenges endanger food systems’ stability and functionality. Effectively addressing these concerns requires developing interdisciplinary research that fosters innovation at the intersections of several disciplines. This approach aims to provide diverse solutions that tackle the social, economic, technical, and policy aspects of these challenges.

## 5. Conclusions

To the best of the authors’ understanding, this study is pioneering in its approach, representing the first systematic review dedicated to an in-depth and comprehensive examination of the diverse and complex impacts of the COVID-19 pandemic on agricultural and food systems, specifically in the NENA region. This research delves into the various dimensions and facets of these impacts, exploring how the pandemic has affected various aspects of agriculture and food systems, ranging from production, supply chain management, and market dynamics to food security and socioeconomic factors within the NENA context.

The analysis of bibliometric data over time shows that the rate of published research outputs on this topic is high and have steadily increased year-over-year. This growing productivity suggests there is heightened interest and focus among scholars and scientists in conducting studies related to this research field. The emerging research findings span diverse areas, indicating their multidisciplinarity. Examining author affiliations highlights active participation from institutions in Saudi Arabia, Egypt, Jordan, the United Arab Emirates, and Lebanon, especially. This potentially signals both the overall vitality and strengths of the research systems within the NENA region, as well as subject-specific research capabilities concentrated in these leading countries. However, mapping the geography and distribution of published research revealing this subject uncovers notable imbalances between different NENA nations. The majority of coverage of existing studies originates from Saudi Arabian institutions, followed by meaningful but lower output from Lebanon, Jordan, and Egypt. Generally, research productivity from wealthy Gulf countries seems higher than that of less developed North African nations or those undergoing severe political unrest and conflicts, such as Libya, Syria, and Yemen.

Concerning agriculture subsectors, an analysis of the selected documents reveals a noticeable trend: the majority, particularly those focusing on shifts in food consumption patterns and dietary habits during the COVID-19 pandemic, lack specificity regarding individual agriculture subsectors. When these studies do address a particular subsector, they predominantly emphasize crop production. In stark contrast, other significant subsectors, such as animal production or livestock and, more notably, fisheries and aquaculture, receive considerably less attention. This gap in research is particularly concerning given that the pandemic has had wide-ranging effects across all stages of the food chain, impacting every step from production to consumption. This includes the processing, transport, and distribution processes, which are integral to the functioning of the food system. However, a closer examination of the selected literature reveals a skewed focus. Most studies primarily explore the downstream facets of the food chain. This includes aspects such as distribution, retail, consumer behavior, and food waste management. These are undoubtedly important areas, but this focus leads to a significant underrepresentation of the upstream elements of the food chain, including the initial production phase. Furthermore, these studies often overlook the intermediate stages of the food chain, which involve crucial processes such as the handling, processing, and packaging of food products. The lack of comprehensive research in these areas presents a critical knowledge gap, considering how integral these stages are in maintaining the resilience and efficiency of food systems, especially in times of global crises like the COVID-19 pandemic. 

The COVID-19 pandemic affected all four pillars of food security (viz., availability, access, use/utilization, and stability). Nevertheless, the scale of the impacts, as well as the level of coverage, change from one pillar to another. In fact, most of the selected documents focus on food access and food utilization. Concerning food access, the COVID-19 pandemic influenced both economic accessibility/affordability and physical accessibility. The containment measures for the virus, such as lockdowns, impacted physical access to food in NENA (Near East and North Africa) countries. Economic access to food was also adversely affected due to rising food prices. Additionally, purchasing power diminished because of job losses and the detrimental effects of the pandemic on people’s livelihoods. As for food utilization, the studies focus mainly on dietary diversity, diet quality, and food safety. 

The COVID-19 pandemic affected all dimensions of the sustainability of agri-food systems. Nevertheless, the lion’s share of the scholarly literature deals with the socioeconomic impacts of the pandemic, especially those linked to food (in)security and health, while environmental and political dimensions are less addressed. Regarding the economic aspect, the COVID-19 pandemic and the consequent movement restrictions and limitations significantly impacted socio-economic activities, including the primary sector (agriculture). This led to job losses and income reductions for numerous households. Regarding the social dimension, most studies address the impacts of the COVID-19 pandemic on health and food (in)security/(mal)nutrition. In general, the pandemic affected the quality of life and lifestyles. Research focused on the policy dimension typically explores the ramifications of virus containment strategies and/or the impacts of governmental actions on the economy and the population’s livelihoods. Additionally, these studies often assess the suitability of existing agri-food policies in the context of insights from the pandemic experience. 

The COVID-19 pandemic exposed the significant vulnerabilities and weaknesses embedded across agricultural and food systems globally. This crisis should be leveraged as an opportunity to fundamentally reimagine and transform the agri-food sector toward bolstering sustainability and resilience. The insights and lessons that have emerged throughout the pandemic response should inform preparations and mitigation strategies for building greater capacity to withstand future pandemics, shocks, and disruptions. Specific priority areas needing attention center on enhancing social safety nets and protection policies for the most vulnerable communities. Near East and North Africa (NENA) countries need to address their heavy reliance on food imports by diversifying import sources and partners, as well as increasing domestic crop production and overall self-sufficiency. Food security preparedness and agri-food sector stability should be critical pillars within contingency planning and recovery frameworks. The reduction in reliance on imports also calls for increasing domestic crop production. It is also essential to pay more attention to the agri-food sector and food security issues in contingency plans and recovery strategies. Additionally, the interconnectedness and complexity revealed through this pandemic highlight the necessity of embracing holistic perspectives and systemic approaches in shaping future agri-food policies, strategies, and research agendas. More interdisciplinary efforts will be critical to enact informed, evidence-based decision-making and facilitate effective, equitable, and sustainable outcomes. Investing in research is paramount for supporting this evolution and dealing with ongoing volatility and instability.

This study is novel and original, particularly in the context of the NENA region, marking it as potentially the first of its kind in these areas. This paper’s originality stems from its comprehensive, systematic approach and geographical focus (covering the whole NENA region), which allow delving into issues related to the COVID-19 pandemic hitherto unexplored in the scholarly literature. Indeed, this systematic review provides several important original contributions to the existing literature on the COVID-19 pandemic. In particular, this study provides scientific evidence on how the pandemic affected food systems and food security in the NENA region. Results are presented in tabular form, which makes comparisons among the countries in the region easier. This, in turn, can foster benchmarking exercises that will allow less-performing NENA countries to upgrade their research and development systems in order to be better prepared for eventual upcoming pandemics. Last but not least, the regional focus of this study makes it particularly useful for scholars interested in similar analyses in other world regions as well as future longitudinal studies on the impacts of the pandemic in the NENA region.

The limitations of this study are similar to those of other systematic reviews [90,92,213,214,215,216,217,218,219]. The limitations pertain to selecting the search terms/keywords and utilizing the Web of Science database for the inquiry. As a result, the article encompasses only publications that are indexed in WoS, thus excluding articles published in non-indexed WoS journals (so most journals without an impact factor) as well as the gray literature, i.e., reports, website articles, newspaper articles, etc.

## Figures and Tables

**Table 1:** COVID-19 Pandemic in the NENA Region.

Country	Confirmed Cases *	Deaths *	Vaccine Doses Administered *	Population (Thousands, 2022)
Algeria	271,945	6881	15,267,442	44,903.22
Bahrain	696,614	1536	3,476,633	1472.23
Egypt	516,023	24,830	112,673,535	110,990.10
Iraq	2,465,545	2537	19,557,364	44,496.12
Jordan	1,746,997	14,122	10,057,975	11,285.87
Kuwait	666,486	2570	8,261,153	4268.87
Lebanon	1,239,904	10,947	5,814,699	5489.74
Libya	507,269	6437	3,739,158	6812.34
Mauritania	63,774	997	4,075,704	4736.14
Morocco	1,277,342	16,297	55,389,602	37,457.97
Oman	399,449	4628	7,086,050	4576.30
Qatar	514,524	690	7,609,178	2695.12
Saudi Arabia	841,469	9646	68,534,631	36,408.82
Sudan	63,993	5046	22,598,737	46,874.20
Syria	57,423	3163	5,090,630	22,125.25
Tunisia	1,153,361	29,423	13,253,317	12,356.12
United Arab Emirates (UAE)	1,067,030	2349	24,922,054	9441.13
Yemen	11,945	2159	128,768	33,696.61
Source	WHO [2]	WHO [2]	WHO [2]	World Bank [10]

* As of 4 November 2023.

**Table 3:** Previous reviews dealt with COVID-19 and agri-food in the NENA region. Source: Authors’ elaboration based on data from the literature.

Review	Publication Date	Review Type	Geographical Coverage	Thematic Focus
Ben Hassen and El Bilali [5]	December 2022	Narrative review	Gulf Cooperation Council (GCC) region	Food security Food consumption patterns
Alshubaith et al. [53]	October 2022	Narrative review	Global Only one NENA country (viz. Saudi Arabia) addressed	Environment sustainability Animal health Food security Food safety
Mignogna et al. [54]	April 2022	Systematic review	Global Only five NENA countries (viz. Iraq, Jordan, Kuwait, UAE and Saudi Arabia) addressed	Food intake Eating behaviours Diet quality
Elloumi [55]	December 2020	Narrative review	Tunisia	Agriculture
Al Jawaldeh et al. [56]	November 2020		Eastern Mediterranean (All NENA countries except Mauritania)	Childhood stunting

**Table 4:** Selection of eligible documents.

Selection Steps	Number of Selected Documents	Number of Excluded Documents and Reasons for Exclusion
Search on WoS	334	21 documents published before the outbreak of SARS-CoV-2 (cf. COVID-19)
Screening of documents based on titles	313	16 documents were excluded because they deal with countries outside the NENA region, e.g., Australia, Bangladesh, Ethiopia, Indonesia, Iran, Nigeria, Pakistan, the Philippines, Romania, South Korea, Spain, Turkey, Uganda, and the USA
Screening of documents based on abstracts	297	139 documents were excluded: 35 documents that do not deal with NENA countries 32 documents that do not deal with COVID-19 69 documents that do not address agri-food systems 3 editorial materials
Scrutiny of full-text	158	7 documents were excluded: 1 document that does not deal with NENA 1 document that does not address agri-food 5 reviews
Confirmation of eligibility and inclusion in the systematic review	151	--

**Table 5:** Specific Target Groups in the Selected Studies.

Examples of the Specific Target Groups	Documents
Children and adolescents/teens	Al Agha et al. [60]; Alfayez et al. [61]; Alghadir et al. [62]; Al-Mulla and Mahfoud [63]; Bahatheg [64]; Benmerzoug et al. [65]; Butt et al. [66]; Gedeon et al. [67]; Hanbazaza and Wazzan [68]; Hoteit et al. [69]; Kamaleddine et al. [70]; Oakley et al. [71]; Sajwani et al. [72]
Women (including pregnant, post-partum, and lactating women)	Algheshairy et al. [73]; Almanasrah et al. [74]; Al-Musharaf [75]; Al-Musharaf et al. [76]; Arfaoui and Alghafari [77]; Ben Hassen et al. [8]; Bossenbroek and Ftouhi [78]; Bouzidi and Abdellaoui [79]; El-Akabawy et al. [80]; Hoteit et al. [81]; Mosli et al. [82]; Osaili et al. [83]; Tayyem et al. [84]; Tayyem et al. [85]
Patients and sick people	Ahmed [86]; Al Kassaa et al. [87]; Helal et al. [88]; Tayyem et al. [89]

**Table 6:** Analyses undergone by the eligible documents.

Item Addressed	Description	Used Method Reference
Bibliographical metrics	Sources/journals/publication titles, research areas, authors, affiliation institutions/organizations, and countries/regions	El Bilali [90] and El Bilali et al. [91]
Research geography	NENA countries where studies were performed	El Bilali [90]; El Bilali and Ben Hassen [92] and El Bilali et al. [91]
Agriculture subsectors	Crop production (and main crops addressed), animal/livestock production, and fisheries/aquaculture	El Bilali [90] and El Bilali et al. [91]
Food chain stages	Production, processing, distribution/retail/marketing, and consumption (including waste management)	El Bilali [90] and El Bilali et al. [91]
Food security	Food security dimensions/pillars: availability, access, utilization/use, and stability	El Bilali [93] and El Bilali [90]
Sustainability	Sustainability dimensions: environment, economy, society, and policy and governance	El Bilali et al. [22]

**Table 10:** Sustainability of the NENA agri-food system in the context of the COVID-19 pandemic. Source: Authors.

Sustainability Dimension *	Documents	Topics Addressed
Environment	Abualhaija and Shammout [111]; Ali and Gad [100]; Al-Saidi and Hussein [95]; Batisha [101]; Ftouhi et al. [201]; Rachidi et al. [136]; Saidi et al. [137]; Saidi et al. [138]; Samara et al. [192]; Selim and Eltarabily [107]; Sraïri [139]	Energy Land Waste Water
Economy	Abu Hatab et al. [98]; Abu Hatab et al. [99]; Al Sadig et al. [150]; Alalwan et al. [195]; Alazaiza et al. [140]; Al-Doori et al. [109]; Algheshairy et al. [73]; Almutairi [161]; Alsuwailem et al. [165]; Batisha [101]; Belton et al. [208]; Ben Hassen et al. [145]; Ben Hassen et al. [125]; Ben Hassen et al. [6]; Ben Hassen et al. [141]; Bossenbroek and Ftouhi [78]; Bouzidi and Abdellaoui [79]; Dimassi et al. [127]; El Bilali et al. [135]; Elgammal et al. [171]; El-Haddad and Zaki [102]; Faour-Klingbeil et al. [206]; Gedeon et al. [67]; Hamade [130]; Hesham et al. [174]; Kaitibie et al. [146]; Koussani and Khamassi [184]; Labidi [185]; Lafta and Mawlood [110]; Mansour et al. [142]; Olaimat et al. [116]; Saidi et al. [137]; Saidi et al. [138]; Saleh [123]; Sobaih and Moustafa [179]; Sundarakani and Onyia [193]; YahiaMarzouk and Jin [108]; Zainal et al. [124]	Employment Financial management Income Jobs Market/marketing Poverty Prices of inputs and products
Society and culture	Abduljawad [147]; Abolfotouh et al. [148]; Abouzid et al. [96]; Abu Hatab et al. [99]; Ahmed [86]; Aijehany and Allily [149]; Al Agha et al. [60]; Al Kassaa et al. [87]; Al-Abdi et al. [143]; Alafif et al. [151]; Alah et al. [144]; Alamri et al. [152]; Alazaiza et al. [140]; AlBlooshi et al. [188]; Aldhwayan and Alabdulkader [153]; Alfayez et al. [61]; Alghadir et al. [62]; Algheshairy et al. [73]; Alhaffar et al. [182]; Alharthi [154]; Alhusseini et al. [156]; Ali et al. [189]; Alkhalaf et al. [157]; Alkhaldy et al. [158]; ALkharashi [159]; Almanasrah et al. [74]; Almousa and Alagal [160]; Al-Mulla and Mahfoud [63]; Al-Musharaf [75]; Al-Musharaf et al. [76]; Almutairi [161]; Alothman et al. [162]; Alotiby and Al-Harbi [163]; Alouani et al. [199]; Alqurashi [164]; Al-Saidi and Hussein [95]; Al-Sejari and Al-Ma’Seb [120]; AlTarrah et al. [121]; Alyami et al. [166]; Ammar et al. [207]; Arfaoui and Alghafari [77]; Azazz and Elshaer [167]; Bahatheg [64]; Bakhsh et al. [168]; Batisha [101]; Ben Hassen et al. [145]; Ben Hassen et al. [125]; Ben Hassen et al. [6]; Ben Hassen et al. [141]; Ben Khadda et al. [200]; Benmerzoug et al. [65]; Bossenbroek and Ftouhi [78]; Bouzidi and Abdellaoui [79]; Braiji et al. [169]; Bushnaq et al. [170]; Butt et al. [66]; Chaiban et al. [133]; Cheikh Ismail et al. [126]; Cheikh Ismail et al. [203]; Dimassi et al. [127]; El Bilali et al. [135]; El Khoury and Julien [128]; El-Akabawy et al. [80]; El-Malah et al. [204]; Elsahoryi et al. [112]; Faour-Klingbeil et al. [206]; Faour-Klingbeil et al. [205]; Faour-Klingbeil et al. [94]; Fiddian-Qasmiyeh [129]; Ftouhi et al. [201]; Gedeon et al. [67]; Ghali-Zinoubi [183]; Hammoudi et al. [131]; Hanbazaza [172]; Hanbazaza and Wazzan [68]; Hariri et al. [173]; Helal et al. [88]; Hesham et al. [174]; Hoteit et al. [81]; Hoteit et al. [69]; Hoteit et al. [196]; Husain and Ashkanani [122]; Issa et al. [113]; Jalal et al. [175]; Jawed et al. [176]; Kaitibie et al. [146]; Kamaleddine et al. [70]; Khabour and Hassanein [103]; Khamees et al. [114]; Kharroubi et al. [132]; Kilani et al. [97]; Koussani and Khamassi [184]; Lafta and Mawlood [110]; Mansour et al. [142]; McCall et al. [49]; Mertens and Peñalvo [209]; Mohsen et al. [105]; Mosli et al. [82]; Mumena [177]; Nohra et al. [134]; Nour [106]; Oakley et al. [71]; Olaimat et al. [115]; Olaimat et al. [116]; Osaili et al. [117]; Osaili et al. [83]; Osaili et al. [118]; Pritchard et al. [210]; Rachidi et al. [136]; Radwan et al. [190]; Radwan et al. [191]; Ragetlie et al. [186]; Rahmat et al. [47]; Saaty and Aljadani [178]; Saidi et al. [137]; Saidi et al. [138]; Sajwani et al. [72]; Saleh [123]; Sobaih and Moustafa [179]; Sultan et al. [180]; Takshe et al. [194]; Taybeh et al. [119]; Tayyem et al. [89]; Tayyem et al. [84]; Tayyem et al. [85]; Turki et al. [187]; Woertz [197]; Zakout et al. [181]; Zuntz et al. [198]	Culture Food (in)security and nutrition/malnutrition Food safety Gender Health Lifestyle Livelihoods Migration Resilience Vulnerability
Policy and governance	Alalwan et al. [195]; Alhusseini and Alqahtani [155]; Batisha [101]; Jouili and Elloumi [202]; Marzouk et al. [104]; Sraïri [139]; Woertz [197]	Appropriateness of policy measures Coping and mitigation strategies

* Several documents address different sustainability dimensions.

**Table 11:** Recommendations to make the agri-food system in the NENA region more sustainable and resilient. Source: Authors.

Examples of Recommendations	Sources
Adopting gender-sensitive strategies and policies to address overweight/obesity and dietary diversity during emergent pandemics and shocks	Ben Hassen et al. [8]; Tayyem et al. [85]
Guaranteeing that containment measures are context-specific to be effective and mitigate side effects on the population’s livelihoods	Al-Mulla and Mahfoud [63]; Alhaffar et al. [182]; Nour [106]
Educating and training workers and the population on food safety	Almanasrah et al. [74]; Arfaoui and Alghafari [77]; Faour-Klingbeil et al. [206]; Faour-Klingbeil et al. [94]; Olaimat et al. [115]; Osaili et al. [117]
Encouraging online shopping, e-commerce, and home delivery by upgrading information and communication technology (ICT) and improving internet speed	Ben Hassen et al. [6]
Establishing food safety standards and regulations for home-based food businesses	Osaili et al. [83]
Improving coordination among actors in the supply chain	Saidi et al. [137]
Paying more attention to food and nutrition security to avoid health crises and pandemics becoming humanitarian crises	Ben Hassen et al. [6]
Raising awareness and improving the knowledge of the population about the importance of healthy eating and diets amidst crises	Abduljawad [147]; AlBlooshi et al. [188]; Turki et al. [187]; Alhusseini and Alqahtani [155]; Alhusseini et al. [156]; Alkhalaf et al. [157]; AlTarrah et al. [121]; El-Akabawy et al. [80]; Hoteit et al. [81]; Husain and Ashkanani [122]; Mumena [177]; Radwan et al. [190]
Reducing food import dependency and diversifying import sources	Ali et al. [189]; Jouili and Elloumi [202]
Strengthening digital extension services in rural areas, especially for women	Ragetlie et al. [186]

## Data Availability

Data is contained within the article.

## Appendix A. List of the Selected Eligible Documents

**Year**	**Number of Documents**	**References**
2023 *	8	AlBlooshi et al. [188]; Alkhaldy et al. [158]; El-Haddad and Zaki [102]; Jawed et al. [176]; Kaitibie et al. [146]; Lafta and Mawlood [110]; Osaili et al. [118]; Tayyem et al. [89]
2022	74	Abualhaija and Shammout [111]; Aijehany and Allily [149]; Al Sadig et al. [150]; Al-Abdi et al. [143]; Alazaiza et al. [140]; Aldhwayan and Alabdulkader [153]; Alfayez et al. [61]; Algheshairy et al. [73]; Alhaffar et al. [182]; Alhusseini et al. [156]; Ali and Gad [100]; Ali et al. [189]; Alkhalaf et al. [157]; ALkharashi [159]; Almanasrah et al. [74]; Almousa and Alagal [160]; Al-Mulla and Mahfoud [63]; Alouani et al. [199]; Azazz and Elshaer [167]; Batisha [101]; Ben Hassen et al. [6]; Ben Hassen et al. [141]; Ben Hassen et al. [8]; Ben Khadda et al. [200]; Benmerzoug et al. [65]; Bouzidi and Abdellaoui [79]; Braiji et al. [169]; Bushnaq et al. [170]; Butt et al. [66]; Chaiban et al. [133]; El-Akabawy et al. [80]; Elgammal et al. [171]; El-Malah et al. [204]; Faour-Klingbeil et al. [94]; Gedeon et al. [67]; Hariri et al. [173]; Helal et al. [88]; Hoteit et al. [81]; Hoteit et al. [69]; Hoteit et al. [196]; Jouili and Elloumi [202]; Kamaleddine et al. [70]; Khamees et al. [114]; Koussani and Khamassi [184]; Labidi [185]; Mansour et al. [142]; Marzouk et al. [104]; McCall et al. [49]; Mohsen et al. [105]; Mosli et al. [82]; Nohra et al. [134]; Nour [106]; Oakley et al. [71]; Olaimat et al. [115]; Olaimat et al. [116]; Osaili et al. [83]; Rachidi et al. [136]; Radwan et al. [191]; Ragetlie et al. [186]; Rahmat et al. [47]; Saidi et al. [137]; Saidi et al. [138]; Sajwani et al. [72]; Samara et al. [192]; Selim and Eltarabily [107]; Sobaih and Moustafa [179]; Takshe et al. [194]; Taybeh et al. [119]; Tayyem et al. [84]; Tayyem et al. [85]; Turki et al. [187]; YahiaMarzouk and Jin [108]; Zainal et al. [124]; Zuntz et al. [198]
2021	55	Abduljawad [147]; Abolfotouh et al. [148]; Abouzid et al. [96]; Abu Hatab et al. [98]; Abu Hatab et al. [99]; Al Agha et al. [60]; Al Kassaa et al. [87]; Alafif et al. [151]; Alah et al. [144]; Alalwan et al. [195]; Alamri et al. [152]; Al-Doori et al. [109]; Alghadir et al. [62]; Alharthi [154]; Al-Musharaf et al. [76]; Almutairi [161]; Alothman et al. [162]; Alotiby and Al-Harbi [163]; Alqurashi [164]; Al-Saidi and Hussein [95]; Al-Sejari and Al-Ma’Seb [120]; Alsuwailem et al. [165]; AlTarrah et al. [121]; Arfaoui and Alghafari [77]; Bahatheg [64]; Bakhsh et al. [168]; Belton et al. [208]; Ben Hassen et al. [125]; Bossenbroek and Ftouhi [78]; Cheikh Ismail et al. [126]; Cheikh Ismail et al. [203]; Dimassi et al. [127]; El Bilali et al. [135]; El Khoury and Julien [128]; Faour-Klingbeil et al. [205]; Faour-Klingbeil et al. [206]; Ftouhi et al. [201]; Ghali-Zinoubi [183]; Hamade [130]; Hammoudi et al. [131]; Hanbazaza [172]; Hanbazaza and Wazzan [68]; Hesham et al. [174]; Issa et al. [113]; Jalal et al. [175]; Khabour and Hassanein [103]; Kharroubi et al. [132]; Mertens and Peñalvo [209]; Osaili et al. [117]; Radwan et al. [190]; Saaty and Aljadani [178]; Saleh [123]; Sraïri [139]; Sultan et al. [180]; Sundarakani and Onyia [193]
2020	14	Ahmed [86]; Alhusseini and Alqahtani [155]; Al-Musharaf [75]; Alyami et al. [166]; Ammar et al. [207]; Ben Hassen et al. [145]; Elsahoryi et al. [112]; Fiddian-Qasmiyeh [129]; Husain and Ashkanani [122]; Kilani et al. [97]; Mumena [177]; Pritchard et al. [210]; Woertz [197]; Zakout et al. [181]
* As of 17 March 2023.		
